# Whiteflies Glycosylate Salicylic Acid and Secrete the Conjugate *via* Their Honeydew

**DOI:** 10.1007/s10886-014-0543-9

**Published:** 2015-01-08

**Authors:** Arjen VanDoorn, Michel de Vries, Merijn R. Kant, Robert C. Schuurink

**Affiliations:** 1 Department of Plant Physiology, University of Amsterdam, Science Park 904, 1098 XH Amsterdam, Netherlands; 2 Institute for Biodiversity and Ecosystem Dynamics, University of Amsterdam, Science Park 904, 1098 XH Amsterdam, Netherlands; 3 Present Address: Roche Diagnostics, Instrumental Analytics, Nonnenwald 2, 82377 Penzberg, Germany

**Keywords:** Whitefly, Phloem feeder, Plant defense, Herbivory, Honeydew, Elicitor, Salicylic acid

## Abstract

During insect feeding, a complex interaction takes place at the feeding site, with plants deciphering molecular information associated with the feeding herbivore, resulting in the upregulation of the appropriate defenses, and the herbivore avoiding or preventing these defenses from taking effect. Whiteflies can feed on plants without causing significant damage to mesophyll cells, making their detection extra challenging for the plant. However, whiteflies secrete honeydew that ends up on the plant surface at the feeding site and on distal plant parts below the feeding site. We reasoned that this honeydew, since it is largely of plant origin, may contain molecular information that alerts the plant, and we focused on the defense hormone salicylic acid (SA). First, we analyzed phloem sap from tomato plants, on which the whiteflies are feeding, and found that it contained salicylic acid (SA). Subsequently, we determined that in honeydew more than 80 % of SA was converted to its glycoside (SAG). When whiteflies were allowed to feed from an artificial diet spiked with labeled SA, labeled SAG also was produced. However, manually depositing honeydew on undamaged plants resulted still in a significant increase in endogenous free SA. Accordingly, transcript levels of *PR1a*, an SA marker gene, increased whereas those of *PI-II,* a jasmonate marker gene, decreased. Our results indicate that whiteflies manipulate the SA levels within their secretions, thus influencing the defense responses in those plant parts that come into contact with honeydew.

## Introduction

Plants and herbivorous insects share a long and complex evolutionary history (Labandeira 2007), where plants have developed multi-leveled defense strategies, and insects have evolved equally complex counter-strategies. The perception of herbivory is crucial for the plant to differentiate insect attack from, for example, wounding to prevent unnecessary activation of costly or autotoxic defense mechanisms. Grazing insects such as caterpillars can be recognized by their continuous wounding (Mithöfer et al. 2005), and/or by deposition of salivary fluids on the wounded plant interface (Musser et al. 2005). Components in these fluids can act as an elicitor and specifically activate plant defense responses. A number of elicitors have been identified in different herbivores. These are compounds, often peptides, isolated from the oral secretions (OS) of insects (Alborn et al. 1997; Halitschke et al. 2001; Schmelz et al. 2006), or fluids deposited during oviposition, giving the plant an early warning (Doss et al. 2000), as reviewed in (Alba et al. 2011). Herbivores however, have evolved mechanisms to evade plant defenses for example by manipulating part of the plant’s defense pathway to their own benefit, also through components in the herbivore’s OS (Hogenhout and Bos 2011; Kant et al. 2008; Musser et al. 2005; Sarmento et al. 2011; Weech et al. 2008).

The sweetpotato whitefly *Bemisia tabaci* (Homoptera: *Aleyrodidae*) is a highly polyphageous insect that has been reported to feed from 420 plant species and 74 families worldwide (Greathead 1986), although more recent country-specific studies suggest this number is likely an underestimation (Erdogan et al. 2008; Perring 2001). *B. tabaci* also poses an economic challenge to crops such as cotton, bell pepper, and tomato, mostly because *B. tabaci* is a vector of *Begomo* viruses, which cause major economic damage. *B. tabaci* is a phloem-feeder, and causes minimal damage to plant cells during feeding, unlike aphids that puncture cells during probing (Janssen et al. 1989). To prevent the collapse of a phloem vessel, aphids release proteins into the phloem that prevent calcium-regulated blockage of the vessel, thus protecting their feeding sites (Will et al. 2007). In whiteflies, a number of enzymes have been identified that could play an important role in dealing with the plant’s defense system (Peng et al. 2013; Su et al. 2012). Thus, it is likely that there are complex interactions between plants and phloem feeders, that find their origin in the phloem. This intimate relationship between phloem-feeders and plants has resulted in plant-defense mechanisms that are similar to the mechanisms found in plant-pathogen interactions, where recognition-based genes (such as NBS-LRRs) can confer a high level of resistance (Klingler et al. 2005; Nombela et al. 2003).

However, the interaction between phloem-feeders and plants is not limited to the feeding mechanism alone since many phloem feeders secrete phloem-derived honeydew, a sticky deposit containing mostly sugars. Phloem concentrations of sugars are estimated as high as 0.5–0.8 M (Cernusak et al. 2003). This honeydew (HD), due to its high sugar content, facilitates secondary infections with (black) molds, but it also presents an opportunity for recognition.

It previously has been shown that whiteflies are susceptible to jasmonic acid (JA)-mediated defenses (Zarate et al. 2007), and it has been suggested that whiteflies activate the salicylic acid (SA) pathway, thus preventing the activation of the JA pathway (Zarate et al. 2007) since cross-talk between SA and JA will prevent the JA pathway from being activated (Koornneef and Pieterse 2008). Hence, induction of SA responses by whiteflies may well be adaptive.

Here, we studied the role of whitefly honeydew in the tomato-whitefly interaction, particularly with respect to the presence of SA in the phloem, the metabolism of SA in whiteflies, and the implications of this for the plant’s defense response.

## Methods and Materials

### Insect Rearing and Honeydew Collection

Whiteflies (*B. tabaci* biotype B) were reared in a growth chamber as previously described (Bleeker et al. 2011), on a diet of both cucumber (*Cucumis sativus*) and tomato (*Lycopersicum esculentum* cultivar Moneymaker) plants. For HD collection, Petri dishes were placed under tomato leaflets in the insect rearing chamber for 24 h. Honeydew was subsequently collected with a pipette by adding 500 μl of ddH_2_O to the Petri dishes.

### Plant Treatment and Phytohormone Analysis

To the adaxial side of leaflets of intact plants 20 μl HD (containing 0.02 % Tween-20 to avoid running-off of the liquid and to promote absorption) or 20 μl ddH_2_O (containing 0.02 % Tween-20) were carefully applied. These leaflets were subsequently harvested after 1 h and extracted for SA analysis according to (VanDoorn et al. 2011). Briefly, samples were extracted in ethyl acetate containing deuterated salicylic acid (D4- SA) (C/D/N Isotopes Inc., Pointe-Claire, Quebec, Canada) as internal standard, evaporated and reconstituted in 70 % (*v/v*) MeOH, and analyzed by LC-MS/MS using instrument parameters as described previously (Scala et al. 2013).

Phloem sap was collected using the ‘EDTA’ method (King and Zeevaart 1974). Tomato leaflets were excised from the plant and their petioles put in a Petri dish containing 5 mM EDTA (pH 8) for 1 min, after which they were transferred to a 50 ml tube containing fresh 5 mM EDTA. The tubes were covered with a plastic tray lined with wet paper towels to increase the humidity, and thus minimizing transpiration. After 1 h, the solution was replaced with fresh 5 mM EDTA (30 ml), and subsequently the phloem sap was collected for 10 h. Four samples were collected, each sample from 5 to 6 leaves, representing ~15 g FW.

For analysis of SAG, the protocol of (van den Burg et al. 2010) was adapted as follows: 200 μl of HD or phloem sap were added to 200 μl Na-acetate solution (0.2 mM pH 4.5) containing 1 mg β-glucosidase (Sigma-Alrich), or 200 μl Na-acetate (control). After overnight incubation at 37 °C, the samples were acidified to pH 1 with 20 μl 37 % HCl, and immediately extracted twice with 700 μl ethyl acetate/pentane/2-propanol (50/50/1 v/v/v). The combined extracts were evaporated, reconstituted in 100 % MeOH, and analyzed by LC-MS/MS as described above. The water phase was used for sugar analysis by LC-MS/MS according to (Clarke et al. 2006). Sugars were separated over a Luna NH_2_-column (Phenomenex) using 50 % Acetonitril (containing 0.05 % formic acid) and 50 % ddH_2_O (containing 0.05 % formic acid) with a flow of 0.5 ml min^−1^ for 5 min. Elution times were 1.5 min for hexose and 3.5 min for dihexose. There was no discrimination between isomers such as glucose and fructose.

### Artificial Diet

A 20-ml plastic container with a soft plastic lid was used as a feeding chamber for whiteflies. A hole (approximately 5 × 5 mm) was cut in the lid, and Parafilm stretched over this. The Parafilm had to be stretched maximally for flies to be able to feed through. A drop of artificial diet was placed on the Parafilm, and a second layer of Parafilm was stretched over this. The artificial diet consisted of a 20 % (*w/v*) sucrose solution containing 2 mg ml^−1^ threonine, and 100 μg ml^−1^ D4-SA. Since SA is only slightly soluble in water, it was first dissolved in a small volume of MeOH and subsequently diluted with ddH_2_O to 1 % MeOH (*v/v*).

After 2 days, containers were placed at −80 °C to kill the whiteflies, and HD was collected in ddH_2_O and analyzed as above. Four containers, each containing approximately 30 whiteflies had to be pooled in order to detect D4-SA in the HD.

For the experiment with SA-laced artificial diet, SA artificial diet containing 100 μg ml^−1^ SA and control diet was prepared as above. In order to get enough HD, 2000 flies per treatment had to be used, with approximately 40 flies per cage. After 3 days, flies were killed at −80 °C and honeydew collected. To dissolve the HD, 100 μl were added to the first cage, and transferred to the next to maximize the HD concentration. Total collected volume was 500 μl.

### Gene Expression Analysis

Plants were treated with honeydew as described above, and samples were taken 24 h later. Total RNA was isolated according to the Logspin protocol (Yaffe et al. 2012). One μg RNA was transcribed into cDNA, and RT-qPCR reactions were carried out according to Spyropoulou et al. 2014. Primers were TGGTGGTTCATTTCTTGCAACTAC and ATCAATCCGATCCACTTATCATTTTA for PR-1a (GB: AJ011520), GTACTGCATCTTCTTGTTTCCA and TAGATAAGTGCTTGATGTCCA for PR-P6 (GB: M69248.1), GACAAGGTACTAGTAATCAATTATCC and GGGCATATCCCGAACCCAAGA for PI-II (GB: AY129402.1). For normalization, actin (GB: XM_004235020.1) transcript levels were determined with the following primers: TTAGCACCTTCCAGCAGATGT and AACAGACAGGACACTCGCACT. Samples were measured with 3 technical replicates.

## Results

### SA and SAG Levels in Honeydew and Phloem

**Fig. 1:**
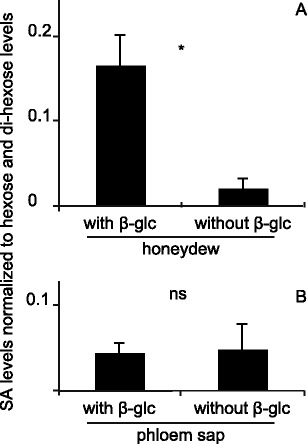
Relative salicylic acid (SA) levels in phloem and honeydew. Honeydew (**a**) and phloem sap (**b**) were collected, treated with and without β-glucosidase, and analyzed by LC-MS/MS for SA and sugar (hexose + dihexose) content. *N* = 4; * indicates a significant difference (Student’s *T*-test, *P* < 0.05); *error bars* represent standard errors

In order to determine the levels of SA and SAG in phloem sap and honeydew using LC/MS, we first collected phloem sap from tomato plants (cultivar Moneymaker) according to a well-established method (King and Zeevaart 1974) and collected honeydew by simply putting a Petri dish under a leaflet infested with whiteflies. Since the methods of collecting phloem sap and honeydew do not allow for a precise determination of their volumes, the levels of SA and SAG were normalized according to their sugar (hexose and dihexose) content. The data show that the ratio of SAG to SA in honeydew is approximately 8 (Fig. 1a) but that the levels of SAG and SA in phloem are approximately equal (Fig. 1b). This indicates that conversion of SA to SAG happens in the whitefly or its honeydew, unless whiteflies metabolize SA and thereby skew the ratio.

### Glycosylation of SA by Whiteflies

**Fig. 2:**
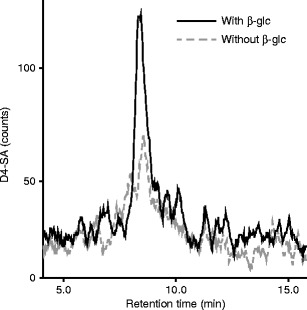
Deuterated salicylic acid (D4-SA) in honeydew after feeding on artificial diet laced with D4-SA. Whiteflies were fed an artificial diet containing D4-SA for 2 d, honeydew (HD) was collected, β-glucosidase treatment performed and subsequently analyzed for D4-SA by LC-MS/MS. The solid, *black line* shows D4-SA in honeydew treated with beta-glucosidase, the dashed grey line without

To investigate the possibility that whiteflies can convert SA to SAG, we fed whiteflies an artificial diet containing D4-labeled SA and sucrose. Subsequently we collected the honeydew derived from this artificial diet, which we retrieved from the bottom of the feeding chambers and analyzed it for SA and SAG levels. The results show that the honeydew indeed contained SAG, *i.e.*, more free D4-SA after β-glucosidase treatment (Fig. 2), but at relatively low absolute levels. This indicated that whiteflies could convert SA to SAG in the presence of sucrose.

### *Effects of Honeydew on SA Levels* in Planta *and on Gene Expression*

**Fig. 3:**
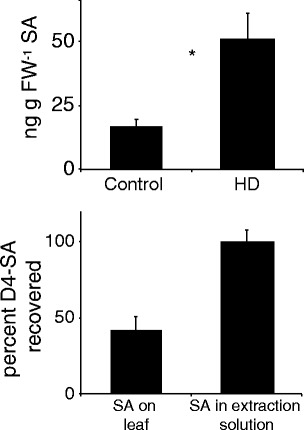
Salicylic acid (SA) levels after honeydew (HD) treatment and recovery of SA. **a** Induction of SA after honeydew treatment. 20 μl honeydew or ddH_2_O (both containing 0.02 % Tween-20) were pipetted onto the adaxial side of a tomato leaf, the leaves were harvested and analyzed for SA and quantified with a deuterated salicylic acid (D4-SA) internal standard. **b** Recovery of SA from the leaf surface. 50 ng SA were applied to the leaf surface and extracted after 1 h. As a control, a non-treated leaf was extracted with the extraction solution containing 50n g D4-SA. *N* = 4 * indicates a significant difference (Student’s *T*-test, *P* < 0.05); *error bars* represent standard errors

To test the effect of honeydew deposition on SA levels, HD (containing approximately 2 ng SA) was applied to undamaged leaves, and the levels of free SA determined after 1 h. The results (Fig. 3a) show that honeydew increased free SA levels to 50 ng g FW^−1^, while the control, water treated, leaves only show a level of 19 ng g-1 FW. To test if applied SA was fully recoverable after application on the leaf surface, 50 ng D4-labeled SA were applied to the leaf surface and extracted. A comparison with the same amount spiked in extraction solution showed that the recovery rate was 42 %(Fig 3b).

**Fig. 4:**
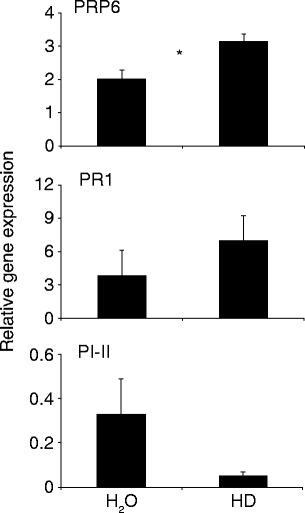
Transcript levels of defense-related genes after honeydew treatment. Plants were treated with 20 μl honeydew or ddH_2_O (both containing 0.02 % Tween-20) and after 24 h the leaf material was harvested for RT-qPCR analyses. Data were normalized to *Actin* transcript levels. *N* = 3, * indicates a statistical difference (Student *T*-test, *P* < 0.05); *error bars* represent standard errors

The increase in SA levels prompted us to study the effects of HD on gene expression, using the *PR-1a* and *PR-P6* genes, well-established SA-markers (van Kan et al. 1992), and the *PI-II* gene, a JA marker (Graham et al. 1985), as read outs. Figure 4 shows that HD application resulted in a moderate increase of *PR1* and *PRP6* transcripts levels over the control treatment, and in a decrease of the *PI-II* transcript levels.

**Fig. 5:**
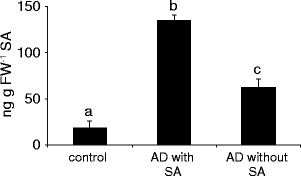
Honeydew from artificial medium laced with salicylic acid (SA) increases SA levels *in planta*. Two thousand whiteflies were fed artificial diet (AD) with or without 100 μg/ml SA for 72 h. The honeydew was subsequently collected, and 20 μl applied to undamaged leaves for 1 h. SA levels were subsequently measured by LC-MS/MS. *Asterisk* indicates a significant difference (ANOVA, followed by Scheffé *posthoc* test *P* < 0.01); *error bars* represent standard errors (*N* = 4)

*Honeydew from SA-fed Whiteflies Induces More SA* in Planta. To test if SA fed through artificial diet influences the SA elicitation in plants, artificial diet was laced with SA. The honeydew was collected and applied to undamaged leaves. As controls, honeydew from SA-free artificial diet and the solvent for honeydew (ddH2O) were used. The results show that lacing whitefly diet with SA leads to increased SA-elicitation *in planta*. Honeydew resulting from artificial diet containing no SA induced SA levels to approximately 60 ng g FW^−1^, whereas SA-laced diet resulted in the elicitation of SA levels to 130 ng g FW^−1^ (Fig. 5).

## Discussion

When herbivores feed, they leave chemical traces behind, in and on plant tissues. Plants can sense and process some of such herbivore-derived signals to activate defense responses. It has been proposed that the mode of action of elicitors takes place *via* ‘self recognition’, where plants recognize (parts of) their own molecules and/or herbivore-specific compounds, sometimes after they have been metabolized by either the plant or the insect (Alborn et al. 1997; Carroll et al. 2008; Heil 2009; Schmelz et al. 2009; VanDoorn et al. 2010). Because phloem feeders excrete a large portion of the fluids they ingest, mostly in the form of small droplets, this provides a ‘recognition opportunity’ for the plant. When we assayed the honeydew of whiteflies as well as their natural diet, tomato phloem sap, for phytohormones, we detected free SA in both (but no JA, data not shown). The presence of SA in phloem had been shown previously (Smith-Becker et al. 1998), while the honeydew of aphids also is know to contain it (Cleland and Ajami 1974). In the honeydew of whiteflies, however, we observed that most of this SA was converted to SAG, a much more polar compound (Figs. 1 and 2). Since depositing free SA on plants will likely elicit a relatively strong defense response, it is possible that the glycosylation of SA to SAG serves as a mechanism to prevent such a strong activation of defense responses in the distal parts of the plant, since this may consume valuable resources for defense or for early flowering, and thus negatively affect resource flows (Agtuca et al. 2013; Argueso et al. 2012; Donovan et al. 2013) and may hence facilitate defense priming rather than direct induction as has been suggested. Salicyclic acid glycoside itself is inactive *in planta* and needs to be converted back to SA to induce SA-related defenses (for a review see *e.g.*, Vlot et al. 2009) An alternative explanation of our results would be direct toxicity of SA to the whiteflies, with glycosylation necessary to detoxify the SA.

When, after honeydew treatment, SA levels *in planta* were analyzed, it appeared that whitefly honeydew slightly induced SA levels (Fig. 3). The level of induction is relatively small, approximately 30 ng g FW^−1^ after 1 h, but this SA could not be contributed to the small levels of SA that are present in honeydew, which are approximately 2 ng in the 20 ul HD applied. Our results are consistent with those of (Schwartzberg and Tumlinson 2014), which showed induction of SA production after application of aphid (*Acyrthosipon pisum*) honeydew to bean (*Vica faba*) plants. However, they did not analyze SAG levels in aphid honeydew, so it is unclear whether aphids also can convert SA to SAG. Moreover, when we applied honeydew resulting from diet with or without SA to leaves, it was clear that honeydew from SA-containing diet induced more SA *in planta* then SA-free diet (Fig. 5). This probably is due to the fact that the SA-containing diet resulted in the presence of SA and SAG in the honeydew, which induced higher SA levels in the plant.

When gene expression after HD application was analyzed, there was a trend towards the induction of genes activated by SA signaling, and towards the suppression of genes activated by JA signaling (Fig. 4), indicating that the small amount of free SA present in the HD is biologically relevant. These results are in line with the vast amount of literature concerning the antagonism of SA on JA signaling in dicots (Koornneef and Pieterse 2008; Spoel and Dong 2008). Moreover, our results also are consistent with the facts that whiteflies feeding on Arabidopsis induce SA-related genes and repress JA-related genes (Zarate et al. 2007), and that whiteflies increase SA-levels when feeding from lima bean plants (*Phaseolus lunatus*) and repress JA-related genes (Zhang *et al.*, 2009). Typically, the density of feeding whiteflies on leaves in these previous experiments was high, up to 200 individuals per Arabidopsis leaf (Zarate et al. 2007) or 50 per bean leaf (Zhang *et al.*, 2009). With these densities, the amount of honeydew deposited will be significant, and thus we argue that the SA and SAG from honeydew of whiteflies could augment salivary factors in relaying defensive processes.

It recently was discovered that aphid (*Acyrthosiphon pisum*) honeydew contains many proteins (from the aphid and associated microbes) that may act as mediators in the plant-aphid interaction (Sabri *et al.*, 2013). This, together with the fact that honeydew of *Acyrthosipon pisum* can attract natural enemies of the aphid (Leroy *et al.*, 2009), and the classical example that ants can harvest the aphid honeydew and in return provide the aphids with protection against predators in some systems (Styrsky and Eubanks 2007; Völkl et al. 1999; Way 1963), all point to a myriad of functions for honeydew in multitrophic systems. Our results, indicating that whiteflies can modulate the SA levels in the honeydew, add to this complexity. Hence, the deposition of honeydew on plants can be an important factor in fine-tuning the interaction between whiteflies and plants, which so far has been overlooked. Whiteflies can feed on a large number of host plants, and the mechanism to modulate SA signaling by glycosylation might be a broad-spectrum mechanism. Further studies will be needed to determine the role of SAG in plant-insect interactions.

## References

[CR1] Agtuca B, Rieger E, Hilger K, Song L, Robert CAM, Erb M, Karve A, Ferrieri RA (2013). Carbon-11 reveals opposing roles of auxin and salicylic acid in regulating leaf physiology, Leaf metabolism, and resource allocation patterns that impact root growth in *Zea mays*. J Plant Growth Regul.

[CR2] Alba JM, Glas JJ, Schimmel BC, Kant MR (2011). Avoidance and suppression of plant defenses by herbivores and pathogens. J Plant Interact.

[CR3] Alborn H, Turlings T, Jones T, Stenhagen G, Loughrin J, Tumlinson J (1997). An elicitor of plant volatiles from beet armyworm oral secretion. Science.

[CR4] Argueso CT, Ferreira FJ, Epple P, To JP, Hutchison CE, Schaller GE, Dangl JL, Kieber JJ (2012). Two-component elements mediate interactions between cytokinin and salicylic acid in plant immunity. PLoS Genet.

[CR5] Bleeker PM, Diergaarde PJ, Ament K, Schütz S, Johne B, Dijkink J, Hiemstra H, de Gelder R, de Both MT, Sabelis MW (2011). Tomato-produced 7-epizingiberene and *R*-curcumene act as repellents to whiteflies. Phytochemistry.

[CR6] Carroll MJ, Schmelz EA, Teal PE (2008). The attraction of *Spodoptera frugiperda* neonates to cowpea seedlings is mediated by volatiles induced by conspecific herbivory and the elicitor inceptin. J Chem Ecol.

[CR7] Cernusak LA, Arthur DJ, Pate JS, Farquhar GD (2003). Water relations link carbon and oxygen isotope discrimination to phloem sap sugar concentration in *Eucalyptus globulus*. Plant Physiol.

[CR8] Clarke MB, Bezabeh DZ, Howard CT (2006). Determination of carbohydrates in tobacco products by liquid chromatography-mass spectrometry/mass spectrometry: a comparison with ion chromatography and application to product discrimination. J Agric Food Chem.

[CR9] Cleland CF, Ajami A (1974). Identification of the flower-inducing factor isolated from aphid honeydew as being salicylic acid. Plant Physiol.

[CR10] Donovan MP, Nabity PD, DeLucia EH (2013). Salicylic acid-mediated reductions in yield in *Nicotiana attenuata* challenged by aphid herbivory. Arthropod-Plant Interactions.

[CR11] Doss RP, Oliver JE, Proebsting WM, Potter SW, Kuy S, Clement SL, Williamson RT, Carney JR, DeVilbiss ED (2000). Bruchins: insect-derived plant regulators that stimulate neoplasm formation. Proc Natl Acad Sci U S A.

[CR12] Erdogan C, Moores GD, Oktay Gurkan M, Gorman KJ, Denholm I (2008). Insecticide resistance and biotype status of populations of the tobacco whitefly *Bemisia tabaci* (Hemiptera: Aleyrodidae) from Turkey. Crop Prot.

[CR13] Graham JS, Pearce G, Merryweather J, Titani K, Ericsson L, Ryan C (1985). Wound-induced proteinase inhibitors from tomato leaves. II. The cDNA-deduced primary structure of pre-inhibitor II. J Biol Chem.

[CR14] Greathead A (1986) *Bemisia tabaci* - a literature survey on the cotton whitefly with an annotated bibliography. C.A.B... International Institute of Biological Control, Cornell University. M.J.W. Cock, series editor.

[CR15] Halitschke R, Schittko U, Pohnert G, Boland W, Baldwin IT (2001). Molecular Interactions between the specialist herbivore *Manduca sexta* (Lepidoptera, Sphingidae) and its natural host *Nicotiana attenuata*. III. Fatty acid-amino acid conjugates in herbivore oral secretions are necessary and sufficient for herbivore-specific plant responses. Plant Physiol.

[CR16] Heil M (2009). Damaged-self recognition in plant herbivore defence. Trends Plant Sci.

[CR17] Hogenhout SA, Bos JI (2011). Effector proteins that modulate plant-insect interactions. Curr Opin Plant Biol.

[CR18] Janssen J, Tjallingii W, Lenteren JV (1989). Electrical recording and ultrastructure of stylet penetration by the greenhouse whitefly. Entomol Exp App.

[CR19] Kant MR, Sabelis MW, Haring MA, Schuurink RC (2008). Intraspecific variation in a generalist herbivore accounts for differential induction and impact of host plant defences. Proc R Soc B.

[CR20] King R, Zeevaart J (1974). Enhancement of phloem exudation from cut petioles by chelating agents. Plant Physiol.

[CR21] Klingler J, Creasy R, Gao L, Nair RM, Calix AS, Jacob HS, Edwards OR, Singh KB (2005). Aphid resistance in *Medicago truncatula* involves antixenosis and phloem-specific, inducible antibiosis, and maps to a single locus flanked by NBS-LRR resistance gene analogs. Plant Physiol.

[CR22] Koornneef A, Pieterse CM (2008). Cross talk in defense signaling. Plant Physiol.

[CR23] Labandeira C (2007). The origin of herbivory on land: Initial patterns of plant tissue consumption by arthropods. Insect Sci.

[CR24] Leroy P, Capella Q, Haubruge E (2009) Aphid honeydew impact on the tritrophic relationships between host-plants, phytophagous insects and their natural enemies. Biotechnol Agron Soc Environ 13:325–334

[CR25] Mithöfer A, Wanner G, Boland W (2005). Effects of feeding S*podoptera littoralis* on lima bean Leaves. II. Continuous mechanical wounding resembling insect feeding is sufficient to elicit herbivory-related volatile emission. Plant Physiol.

[CR26] Musser RO, Cipollini DF, Hum-Musser SM, Williams SA, Brown JK, Felton GW (2005). Evidence that the caterpillar salivary enzyme glucose oxidase provides herbivore offense in solanaceous plants. Arch Insect Biochem Physiol.

[CR27] Nombela G, Williamson VM, Muniz M (2003). The root-knot nematode resistance gene Mi-1.2 of tomato is responsible for resistance against the whitefly *Bemisia tabaci*. Mol Plant Microbe Interact.

[CR28] Peng L, Yan Y, Yang CH, Barro PJ, Wan FH (2013). Identification, comparison, and functional analysis of salivary phenol-oxidizing enzymes in *Bemisia tabaci* B and Trialeurodes vaporariorum. Entomol Exp Appl.

[CR29] Perring TM (2001). The *Bemisia tabaci* species complex. Crop Prot.

[CR30] Sabri A, Vandermoten S, Leroy PD, Haubruge E, Hance T, Thonart P, De Pauw E, Francis F (2013) Proteomic investigation of aphid honeydew reveals an unexpected diversity of proteins. PLoS One. 8(9):e74656. doi: 10.1371/journal.pone.0074656 10.1371/journal.pone.0074656 PMC3783439 24086359

[CR31] Sarmento RA, Lemos F, Bleeker PM, Schuurink RC, Pallini A, Oliveira MGA, Lima ER, Kant M, Sabelis MW, Janssen A (2011). A herbivore that manipulates plant defence. Ecol Lett.

[CR32] Scala A, Mirabella R, Mugo C, Matsui K, Haring MA, Schuurink RC (2013) E-2-hexenal promotes susceptibility to Pseudomonas syringae by activating jasmonic acid pathways in Arabidopsis. Front Plant Sci 4 10.3389/fpls.2013.00074 PMC3624080 23630530

[CR33] Schmelz EA, Carroll MJ, LeClere S, Phipps SM, Meredith J, Chourey PS, Alborn HT, Teal PEA (2006). Fragments of ATP synthase mediate plant perception of insect attack. Proc Natl Acad Sci U S A.

[CR34] Schmelz EA, Engelberth J, Alborn HT, Tumlinson JH, Teal PEA (2009). Phytohormone-based activity mapping of insect herbivore-produced elicitors. Proc Natl Acad Sci U S A.

[CR35] Schwartzberg EG, Tumlinson JH (2014). Aphid honeydew alters plant defence responses. Funct Ecol.

[CR36] Smith-Becker J, Marois E, Huguet EJ, Midland SL, Sims JJ, Keen NT (1998). Accumulation of salicylic acid and 4-hydroxybenzoic acid in phloem fluids of cucumber during systemic acquired resistance is preceded by a transient increase in phenylalanine ammonia-lyase activity in petioles and stems. Plant Physiol.

[CR37] Spoel SH, Dong X (2008). Making sense of hormone crosstalk during plant immune responses. Cell Host Microbe.

[CR38] Spyropoulou EA, Haring MA, Schuurink RC (2014). Expression of Terpenoids 1, a glandular trichome-specific transcription factor from tomato that activates the terpene synthase 5 promoter. Plant Mol Biol.

[CR39] Styrsky JD, Eubanks MD (2007). Ecological consequences of interactions between ants and honeydew-producing insects. Proc R Soc B.

[CR40] Su Y-L, Li J-M, Li M, Luan J-B, Ye X-D, Wang X-W, Liu S-S (2012). Transcriptomic analysis of the salivary glands of an invasive whitefly. PLoS ONE.

[CR41] van den Burg HA, Kini RK, Schuurink RC, Takken FL (2010). Arabidopsis small ubiquitin-like modifier paralogs have distinct functions in development and defense. Plant Cell.

[CR42] van Kan JA, Joosten MH, Wagemakers CA, van den Berg-Velthuis GC, de Wit PJ (1992). Differential accumulation of mRNAs encoding extracellular and intracellular PR proteins in tomato induced by virulent and avirulent races of *Cladosporium fulvum*. Plant Mol Biol.

[CR43] VanDoorn A, Kallenbach M, Borquez AA, Baldwin IT, Bonaventure G (2010). Rapid modification of the insect elicitor N-linolenoyl-glutamate *via* a lipoxygenase-mediated mechanism on *Nicotiana attenuata* leaves. BMC Plant Biol.

[CR44] VanDoorn A, Bonaventure G, Schmidt DD, Baldwin IT (2011). Regulation of jasmonate metabolism and activation of systemic signaling in *Solanum nigrum*: COI1 and JAR4 play overlapping yet distinct roles. New Phytol.

[CR45] Vlot AC, Dempsey DA, Klessig DF (2009). Salicylic Acid, a multifaceted hormone to combat disease. Annu Rev Phytopathol.

[CR46] Völkl W, Woodring J, Fischer M, Lorenz MW, Hoffmann KH (1999). Ant-aphid mutualisms: the impact of honeydew production and honeydew sugar composition on ant preferences. Oecologia.

[CR47] Way MJ (1963). Mutualism between ants and honeydew-producing Homoptera. Annu Rev Entomol.

[CR48] Weech M-H, Chapleau M, Pan L, Ide C, Bede JC (2008). Caterpillar saliva interferes with induced *Arabidopsis thaliana* defence responses *via* the systemic acquired resistance pathway. J Exp Bot.

[CR49] Will T, Tjallingii WF, Thonnessen A, van Bel AJ (2007). Molecular sabotage of plant defense by aphid saliva. Proc Natl Acad Sci U S A.

[CR50] Yaffe H, Buxdorf K, Shapira I, Ein-Gedi S, Zvi MM-B, Fridman E, Moshelion M, Levy M (2012). LogSpin: a simple, economical and fast method for RNA isolation from infected or healthy plants and other eukaryotic tissues. BMC Res Notes.

[CR51] Zarate SI, Kempema LA, Walling LL (2007). Silverleaf whitefly induces salicylic acid defenses and suppresses effectual jasmonic acid defenses. Plant Physiol.

[CR52] Zhang PJ, Zheng SJ, van Loon JJ, Boland W, David A, Mumm R, Dicke M. (2009) Whiteflies interfere with indirect plant defense against spider mites in Lima bean. Proc Natl Acad Sci U S A. 106(50):21202–7 10.1073/pnas.0907890106 PMC2795486 19965373

